# Supplementary material for: In vitro reconstitution of epigenetic reprogramming in the human germ line
Source: Nature. 2024 May 20;631(8019):170–8. doi: 10.1038/s41586-024-07526-6 (PMC11222161; doi:10.1038/s41586-024-07526-6)
Supplement: Supplementary file 1 — This file contains Supplementary Figs. 1–3 and Supplementary Discussions 1 and 2. [file 41586_2024_7526_MOESM1_ESM.pdf]

nature portfolio

https://doi.org/10.1038/s41586-024-07526-6

Supplementary information

In vitro reconstitution of epigenetic reprogramming in the human germ line

In the format provided by the authors and unedited

In vitro reconstitution of epigenetic reprogramming in the human germ line

Yusuke Murase,[1,2,#] Ryuta Yokogawa,[1,2,#] Yukihiro Yabuta,[1,2] Masahiro Nagano,[1,2] Yoshitaka Katou,[1,2] Manami Mizuyama,[1,2] Ayaka Kitamura,[1,2] Pimpitcha Puangsricharoen,[1,2] Chika Yamashiro,[2] Bo Hu,[1,2] Ken Mizuta,[1,2] Kosuke Ogata,[3] Yasushi Ishihama,[3] and Mitinori Saitou[1,2,4]

[1]Institute for the Advanced Study of Human Biology (ASHBi), Kyoto University, Yoshida-Konoe-cho, Sakyo-ku, Kyoto 606-8501, Japan.
[2]Department of Anatomy and Cell Biology, Graduate School of Medicine, Kyoto University, Yoshida-Konoe-cho, Sakyo-ku, Kyoto 606-8501, Japan.
[3]Department of Molecular Systems BioAnalysis, Graduate School of Pharmaceutical Sciences, Kyoto University, Kyoto 606-8501, Japan.
[4]Center for iPS Cell Research and Application (CiRA), Kyoto University, 53 Kawahara-cho, Shogoin, Sakyo-ku, Kyoto 606-8507, Japan.
[#]These authors contributed equally to this work.

**Correspondence should be addressed to:*
Mitinori Saitou, M.D., Ph.D.
E-mail: saitou@anat2.med.kyoto-u.ac.jp
Tel: +81-75-753-4335; Fax: +81-75-751-7286 (MS)

Table of Contents

Supplementary Figure 1. Validation of the AG, DT, and VT reporters.

An orthogonal validation of the expression of fluorescent reporters (AG, DT, VT) during BMP-driven hPGCLC differentiation by immunofluorescence analysis.

Supplementary Figure 2. FACS gating used in this study.

The gating for fluorescence activated cell sorting (FACS) used in this study is shown.

Supplementary Figure 3. Uncropped data for Extended Data Fig. 5i

Uncropped data of Western blot analysis for Extended Data Fig. 5i.

Supplementary Discussion 1.

Discussion on the activities of fluorescent reporters (AG, DT, VT) during BMP-driven hPGCLC differentiation.

Supplementary Discussion 2.

Discussion on the mechanism of epigenetic reprogramming during mouse and human PGC(LC) differentiation.

Supplementary Tables, see the separate Excel documents

Supplementary Table 1. Expression matrix of bulk RNA-seq.

Gene expression matrix of the bulk RNA-seq data presented in Fig. 2 and Fig. 4.

Supplementary Table 2. Overview of bulk RNA-seq, scRNA-seq, EM-seq, ONT long-read-seq samples.

Overview of the samples used for bulk RNA-seq, scRNA-seq, EM-seq, ONT long-read-seq analyses in this study.

Supplementary Table 3. List of DEGs between BMP-driven and xrOvary-based hPGCLC differentiation, and of HVGs in $AG^{+}DT^{-}$ cells cultured with or without BMP2, and their associated GO terms.

List of differentially expressed genes (DEGs) between BMP-driven and xrOvary-based hPGCLC differentiation (Extended Data Fig. 5c), and of highly variable genes (HVGs) in $AG^{+}DT^{-}$ cells cultured with or without BMP2 (Extended Data Fig. 5d–g), and their associated GO terms. *p*-values are provided by Fisher's exact test.

Supplementary Table 4. Lists of DEGs between germ cells *in vivo* and *in vitro* in EM, M or PLL stages, and associated GO terms.

Lists of differentially expressed genes (DEGs) between germ cells *in vivo* and *in vitro* in

early mitotic (EM), mitotic (M) or pre-leptotene (PLL) stages (Extended Data Fig. 6d), and their associated GO terms. *p*-values are provided from Fisher's exact test.

Supplementary Table 5. Statistic values of EM-seq analysis.
Average 5mC methylation levels of relevant genomic elements and of gene promoters [900 bp upstream and 400 bp downstream of transcription start sites (TSS)] in autosomes and X chromosomes in relevant cell types analyzed in this study (Fig. 3 and Extended Data Fig. 7−9).

Supplementary Table 6. Haplotype phasing of chromosome X.
Results of the haplotype phasing of the X chromosomes of F1- and F2-AGVT hiPSCs by ONT long-read-sequence.

Supplementary Table 7. Composition of DNA demethylation escapees.
Composition of the DNA demethylation escapees in the relevant cell types analyzed in this study (Fig. 3 and Extended Data Fig. 8).

Supplementary Table 8. Allelic expression and promoter DNA methylation of X-linked genes, and demethylation escapees.
Allelic expression and promoter DNA methylation of X-linked genes, and demethylation escapees in the relevant cell types analyzed in this study (Extended Data Fig. 9e).

Supplementary Table 9. List of DEGs between wild-type and *TET1* KO cells, and associated GO terms.
List of differentially expressed genes (DEGs) between wild-type and *TET1* KO cells during hPGCLC induction and differentiation, and their clustering and associated GO terms. *p*-values are provided from Fisher's exact test.

Supplementary Table 10. Information of cytokines, chemicals, and culture media.
List of cytokines, chemicals, their concentrations, and culture media used in this study (Extended Data Fig. 1).

Supplementary Table 11. DNA oligonucleotides used in this study.
Sequence information of DNA oligonucleotides for genotyping, reporter construction, knockout by CRISPR, and qPCR used in this study.

Supplementary Table 12. Information of parameters for quality filtering.
Summary of samples, sequencers, quality control filters, and sequence data of the scRNA-seq in this study (Fig. 2c−f, Extended Data Fig. 6).

Supplementary Figure 1. Murase et al.

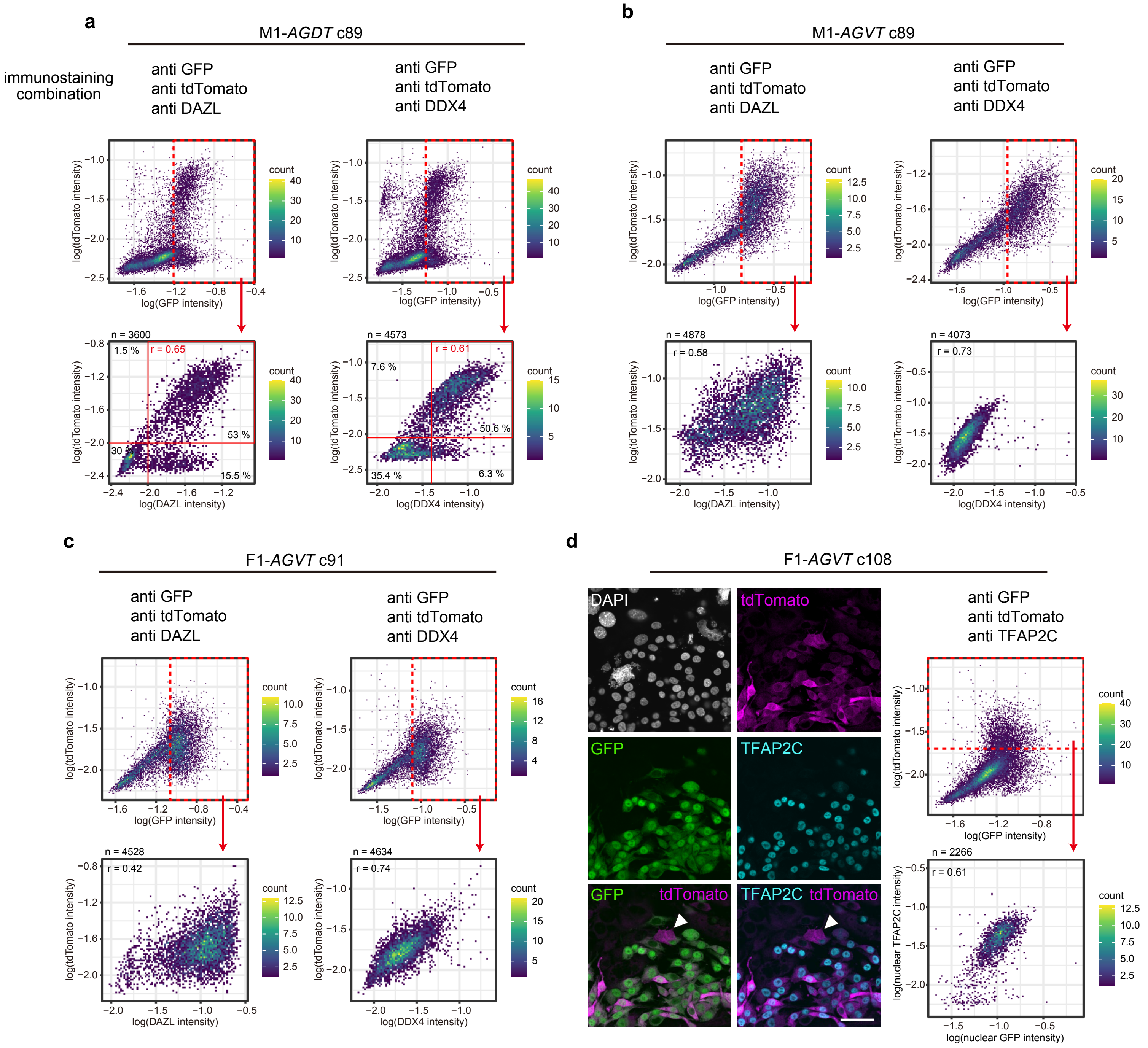

Supplementary Figure 1. Validation of the AG, DT, and VT reporters.
a, IF analysis of GFP, tdTomato, DAZL, and DDX4 expression in M1-*AGDT* hPGCLC-derived cells at c89. (top) Scatter-plot representations of the expression levels [log(fluorescence intensity)] of GFP (AG) and tdTomato (DT) in the c89 cells stained with anti-GFP, anti-tdTomato, and anti-DAZL (left) or anti-DDX4 (right) antibodies. Red-dotted boxed areas were determined as GFP+ (AG+). GFP− and tdTomato− cells include m220 feeders.
(bottom) Scatter-plot representations of the expression levels [log(fluorescence intensity)] of DAZL (left) or DDX4 (right) and tdTomato (DT) in the GFP+ (AG+) cells. Red vertical and horizontal bars indicate a threshold for the DAZL (left) or DDX4 (right) and tdTomato (DT) positivity, respectively. The color coding is as indicated.
b, IF analysis of GFP, tdTomato, DAZL, and DDX4 expression in M1-*AGVT* hPGCLC-derived cells at c89. Scatter-plot representations are as in (**a**).
c, IF analysis of GFP, tdTomato, DAZL, and DDX4 expression in F1-*AGVT* hPGCLC-derived cells at c91. Scatter-plot representations are as in (**a**).
d, IF analysis of GFP, tdTomato, and TFAP2C expression in F1-*AGVT* hPGCLC-derived cells at c108. (left) Representative images for GFP, tdTomato, and TFAP2C expression and their merges as indicated. All AG−VT+ (GFP−tdTomato+) cells (18 cells) were TFAP2C− (arrowheads). Bar, 50 µm. (right) Scatter-plot representations are as in (**a**). Among VT+ cells, GFP (AG) and TFAP2C expression levels were highly correlated (r = 0.61). The color coding is as indicated.

Supplementary Figure 2. Murase et al.

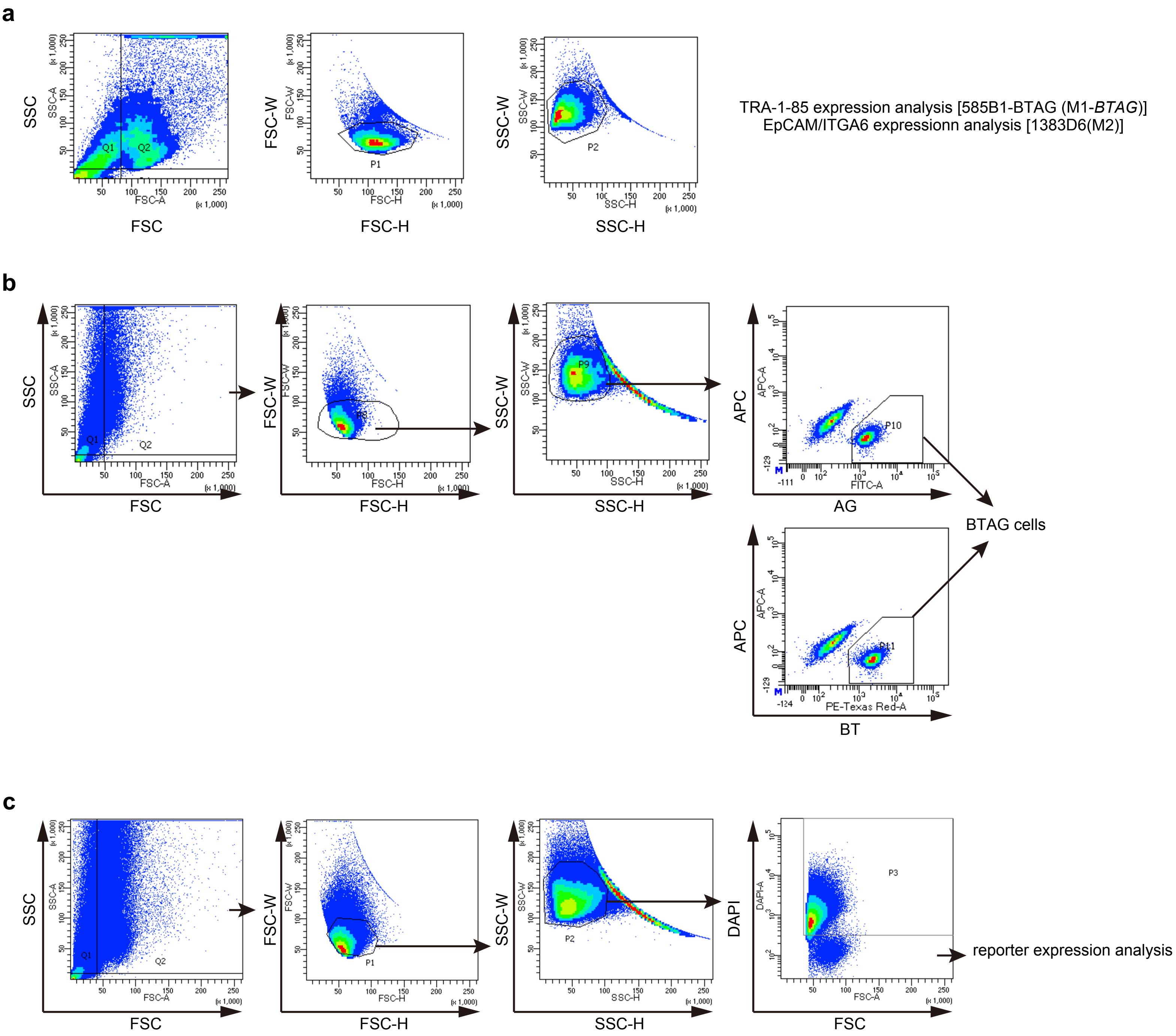

Supplementary Figure 2. FACS gating used in this study.

a, Identification of the cell population used for surface antigen expression analysis in hPGCLC expansion/differentiation culture experiments using 585B1-BTAG (M1-*BTAG*) or 1383D6 (M2) lines.

b, Identification of BTAG cells in hPGCLC expansion/differentiation culture experiments using 585B1-BTAG (M1-*BTAG*) or *TET1* KO lines.

c, Identification of the cell population used for the reporter expression analysis in hPGCLC expansion/differentiation culture experiments using 585B1-AGDT/AGVT (M1-*AGDT*/*AGVT*), NCLCN-AGVT (F1-*AGVT*), and 1390G3-AGVT (F2-*AGVT*) lines.

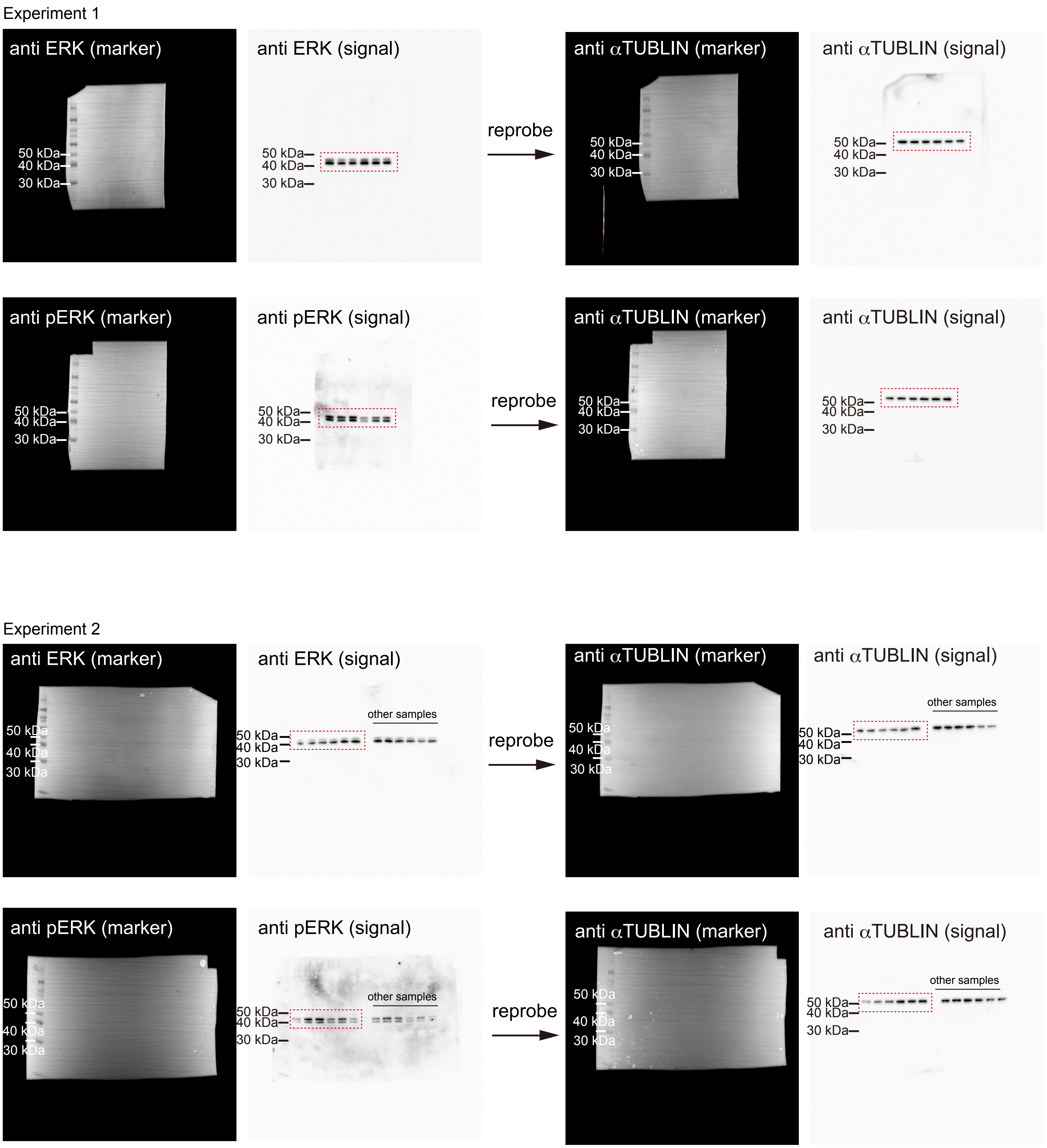

Supplementary Figure 3. Uncroped data for Extended Data Figure. 5i.
Uncropped images of the Western blot analysis shown in Extended Data Fig. 5i of the levels of phosphorylated or total ERK1 and 2 in M1-AGDT hPGCLC-derived cells at c33 cultured with or without BMP2. Three independent cultures were analyzed for two biological replicates (Experiment 1 and 2). The light dotted boxes indicate the regions of interest. αTUBLIN was used for the loading control. pERK: phosphorylated ERK.

Supplementary Discussion 1

We performed an orthogonal validation of the DT and VT reporters. First, we conducted an IF analysis of DAZL and DDX4 expression in M1-*AGDT* hPGCLC-derived cells at c89. This revealed that 1) essentially all DT^+ cells were $DAZL^+$, with the expression levels of DT and DAZL showing an excellent correlation (r = ~0.65); 2) on the other hand, ~one-third of $DT^−$ cells exhibited low/middle-level DAZL positivity; and 3) essentially all $DAZL^−$ cells were $DT^−$ (Supplementary Figure 1a). In accord with these findings, 4) essentially all DT^+ cells were $DDX4^+$, with the expression levels of DT and DDX4 showing a strong correlation (r = ~0.61); 5) a fraction (~15%) of $DT^−$ cells exhibited low/middle-level DDX4 positivity; and 6) the vast majority of all $DDX4^−$ cells were $DT^−$ (Supplementary Figure 1a). These findings demonstrate that the DT positivity is a powerful quantitative indicator for DAZL (and DDX4) expression, while on the other hand, the $DT^−$ cells (at a late stage) include a fraction of DAZL- (and DDX4-) expressing cells at low/middle levels, which may be due to a sporadic selective transcriptional/post-transcriptional silencing of the DT allele during BMP-driven hPGCLC differentiation.

Next, we performed IF analysis of DAZL and DDX4 expression in M1-*AGVT* hPGCLC-derived cells at c89. This revealed that 1) the expression levels of VT and DDX4 were highly correlated in all expression-level ranges (r = ~0.73) and 2) DAZL was broadly expressed from $VT^{−/low}$ to VT^{high} cells, with the expression levels of VT and DAZL showing a mild correlation (r = ~0.58) (Supplementary Figure 1b). These findings demonstrate that VT is a faithful reporter for DDX4 expression, and are consistent with the notion that DAZL begins to be expressed earlier than VT. The IF analysis for F1-*AGVT* hPGCLC-derived cells at c91 gave essentially the same results (Supplementary Figure 1c).

Furthermore, we performed IF analysis of TFAP2C and DDX4 expression in F1-*AGVT* hPGCLC-derived cells at c109. This revealed that 1) essentially all AG^+ cells were $TFAP2C^+$ and 2) all $AG^−VT^+$ cells we detected were $TFAP2C^−$ and $DDX4^+$ (Supplementary Figure 1d), demonstrating that AG is also a faithful reporter for TFAP2C expression.

Collectively, these findings demonstrate that both DT and VT positivity monitor DAZL and DDX4 expression in a highly quantitative manner, while care should be taken for $DT^−$ cells, which include a fraction of $DAZL^+$ (and $DDX4^+$) cells, although the majority are indeed $DAZL^−$ (and $DDX4^−$). Accordingly, we assume that the detection of a relatively high level of *DDX4* in one, but not the other, replicate for the c82 $AG^+DT^−$ cells (Extended Data Fig. 4q) was due to a relatively large proportion of *DDX4*-expressing cells in the former, and that the detection of *DDX4* at a low level in $AG^+VT^−$ cells at c44, c65, and c86 and of *TFAP2C* at a low level in the $AG^−VT^+$ cells at c107 (Fig. 2a) was due to an inclusion of VT^{low} and AG^{low} cells, respectively, upon FACS of the $AG^+VT^−$ and

$AG^{-}VT^{+}$ cell population (Fig. 1c) [note also that the "yield" mode for FACS inevitably sorts in a fraction of non-gated cells].

Supplementary Discussion 2

Mouse germ cells reduce their 5mCs from ~75% to ~5% over a week with an expansion of ~2^9-fold (from ~40 to ~25,000 cells) [97-99], diminishing their 5mCs by ~8% per cell cycle on average. In contrast, human germ cells reduce their 5mCs from ~80% to ~5% over ~5 weeks with an expansion of ~2^{10}-fold (from ~40 to ~40,000 cells) [3,100-102], decreasing their 5mCs by ~7.5% per cell cycle on average. Thus, mouse and human germ cells reduce 5mCs at a similar rate per cell cycle, and accordingly, the difference in time scale for genome-wide DNA demethylation between mice and humans might simply reflect the difference in the doubling time between mouse (~0.8 days on average) and human germ cells (~3.5 days on average). This would further support the notion of a replication-dependent, passive mechanism, which is non-species specific, as the primary mechanism underlying genome-wide DNA demethylation. Unlike mPGCLCs, which reduce their 5mCs by ~10% per cell cycle on average [103], hPGCLC-derived cells from 3 out of 4 hiPSC lines reduce their 5mCs at a lower rate (~3−5%) per cell cycle (Extended Data Fig. 9l), indicating that the hPGCLC differentiation condition would necessitate additional optimization, including the provision of additional factors. Indeed, *UHRF1* remained at a slightly higher level during BMP-driven hPGCLC differentiation than during *in vivo* hPGC differentiation (Fig. 2a), and the former requires substantial propagation for genome-wide demethylation. Given that BMP-driven hPGCLC differentiation displays an attenuation of the MAPK/ERK signalling (Extended Data Fig. 5g−j), a fine tuning of the balance between BMP and MAPK/ERK signalling could be a key for such optimization. On the other hand, hPGCLC-derived cells from F2-*AGVT* reduce their 5mCs by ~7.6% per cell cycle, but with a prolonged doubling time (~8.8 days) (Extended Data Fig. 9l), which might be due to a variance of signalling efficiency associated with this line.

97 Tam, P. P. & Snow, M. H. Proliferation and migration of primordial germ cells during compensatory growth in mouse embryos. *J Embryol Exp Morphol* **64**, 133-147 (1981).
98 Seisenberger, S. *et al.* The dynamics of genome-wide DNA methylation reprogramming in mouse primordial germ cells. *Mol Cell* **48**, 849-862 (2012). https://doi.org/10.1016/j.molcel.2012.11.001
99 Kagiwada, S., Kurimoto, K., Hirota, T., Yamaji, M. & Saitou, M. Replication-coupled passive DNA demethylation for the erasure of genome imprints in mice. *EMBO J* **32**, 340-353 (2013). https://doi.org/10.1038/emboj.2012.331
100 Gkountela, S. *et al.* DNA Demethylation Dynamics in the Human Prenatal Germline. *Cell* **161**, 1425-1436 (2015). https://doi.org/10.1016/j.cell.2015.05.012
101 Guo, F. *et al.* The Transcriptome and DNA Methylome Landscapes of Human Primordial Germ Cells. *Cell* **161**, 1437-1452 (2015). https://doi.org/10.1016/j.cell.2015.05.015
102 Mamsen, L. S., Lutterodt, M. C., Andersen, E. W., Byskov, A. G. & Andersen, C. Y. Germ cell numbers in human embryonic and fetal gonads during the first two trimesters of pregnancy: analysis of six published studies. *Hum Reprod* **26**, 2140-2145 (2011). https://doi.org/10.1093/humrep/der149
103 Ohta, H. *et al.* In vitro expansion of mouse primordial germ cell-like cells recapitulates an epigenetic blank slate. *EMBO J* **36**, 1888-1907 (2017). https://doi.org/10.15252/embj.201695862
