# Supplementary material for: In vitro reconstitution of epigenetic reprogramming in the human germ line
Source: Nature. 2024 May 20;631(8019):170–8. doi: 10.1038/s41586-024-07526-6 (PMC11222161; doi:10.1038/s41586-024-07526-6)
Supplement: Supplementary file 2 — Reporting Summary [file 41586_2024_7526_MOESM2_ESM.pdf]

nature portfolio

Corresponding author(s): Mitinori Saitou

Last updated by author(s): Apr 16, 2024

Reporting Summary

Nature Portfolio wishes to improve the reproducibility of the work that we publish. This form provides structure for consistency and transparency in reporting. For further information on Nature Portfolio policies, see our Editorial Policies and the Editorial Policy Checklist.

Statistics

For all statistical analyses, confirm that the following items are present in the figure legend, table legend, main text, or Methods section.

n/a	Confirmed	
☐	☒	The exact sample size (*n*) for each experimental group/condition, given as a discrete number and unit of measurement
☐	☒	A statement on whether measurements were taken from distinct samples or whether the same sample was measured repeatedly
☐	☒	The statistical test(s) used AND whether they are one- or two-sided<br>*Only common tests should be described solely by name; describe more complex techniques in the Methods section.*
☒	☐	A description of all covariates tested
☐	☒	A description of any assumptions or corrections, such as tests of normality and adjustment for multiple comparisons
☐	☒	A full description of the statistical parameters including central tendency (e.g. means) or other basic estimates (e.g. regression coefficient) AND variation (e.g. standard deviation) or associated estimates of uncertainty (e.g. confidence intervals)
☐	☒	For null hypothesis testing, the test statistic (e.g. *F*, *t*, *r*) with confidence intervals, effect sizes, degrees of freedom and *P* value noted<br>*Give P values as exact values whenever suitable.*
☒	☐	For Bayesian analysis, information on the choice of priors and Markov chain Monte Carlo settings
☒	☐	For hierarchical and complex designs, identification of the appropriate level for tests and full reporting of outcomes
☐	☒	Estimates of effect sizes (e.g. Cohen's *d*, Pearson's *r*), indicating how they were calculated

Our web collection on statistics for biologists contains articles on many of the points above.

Software and code

Policy information about availability of computer code

Data collection	CFX384 Touch Real-Time PCR detection system (Bio-Rad Laboratories)<br>PromethION Flow Cells R9.4.1 (Oxford Nanopore Technologies)<br>PromethION 24 sequencer (Oxford Nanopore Technologies)<br>Novaseq 6000 sequencer (Illumina)<br>Nextseq 500/550 sequencer (Illumina)<br>CKX41 inverted microscope (Olympus)<br>DS-Fi2 microscopic camera (Nikon)<br>M205C microscope (Leica)<br>DP72 microscopic camera (Olympus)<br>FV1000-IX81 confocal microscope system (Olympus)<br>BZX810 (Keyence)<br>Orbitrap Fusion Lumos (Thermo Fisher Scientific)<br>Ultimate 3000 pump (Thermo Fisher Scientific)<br>HTC-PAL autosampler (CTC analytics)<br>Fusion solo S (VILBER LOURMAT)
Data analysis	FV10-ASW v4.1 (Olympus)<br>FACSDiva v8.0.2 (BD)<br>LiftOver program (https://genome.ucsc.edu/cgi-bin/hgLiftOver)<br>Fiji (ImageJ v1.52i)<br>CaptAdvance v16.13b (VILBER LOURMAT)

FragPipe v20.0 (MSFragger v3.8, Philosopher v5.0.0, IonQuant v1.9.8)
Excel 2010 (Microsoft)
R version v4.0.3
DAVID 2021 (https://davidbioinformatics.nih.gov)
RECODE v1.1.1
Cellranger v6.0.1
cutadapt v1.18 / v1.9.1
Tophat v2.1.1
Bowtie2 v2.3.4.1
HTSeq v0.9.1
cuffinks v.2.2.1
Trim galore! v0.4.1 / v0.6.3
Bismark v0.22.1
Samtools v1.15.1 / v1.7
Methpipe v3.4.3
bedtools v2.29.2
Homer v4.11.1
deepTools v3.5.0
PicardTools v2.18.23
MACS2 v2.1.1
Megalodon v2.5.0
Clair3 v0.1.12
bcftools v1.15.1
Whatshap v1.4
modbam2bed v0.5.3
SNPsplit v0.3.2
scVelo v0.2.5
scanpy v1.9.1
Scrublet v0.2.3
screcode v0.1.2
DiffBind v3.8.4
cuML v23.02.00
Seurat v4.2.1 / v4.1.1
stats v3.6.1
ggplot2 v3.4.1
rstatix v0.7.2
SimComp v3.3
ggsignifr v0.6.0
ChIPpeakAnno v3.24.2

For manuscripts utilizing custom algorithms or software that are central to the research but not yet described in published literature, software must be made available to editors and reviewers. We strongly encourage code deposition in a community repository (e.g. GitHub). See the Nature Portfolio guidelines for submitting code & software for further information.

Data

Policy information about availability of data

All manuscripts must include a data availability statement. This statement should provide the following information, where applicable:

- Accession codes, unique identifiers, or web links for publicly available datasets
- A description of any restrictions on data availability
- For clinical datasets or third party data, please ensure that the statement adheres to our policy

Human genome reference GRCh38.p12(https://www.ncbi.nlm.nih.gov/datasets/genome/GCF_000001405.38/) and GRCh38.p2(https://www.ncbi.nlm.nih.gov/datasets/genome/GCF_000001405.28/) are used in this study. The accession number for the sequencing data (bulk RNA-seq, scRNA-seq, EM-seq) generated in this study is GSE232078 (the GEO database). The R script is available on request. ONT long read sequence data have been deposited to NBDC Human Database with accession number JGAS000690. The raw MS data and analysis files have been deposited in the ProteomeXchange Consortium (http://proteomecentral.proteomexchange.org) via the jPOST partner repository (https://jpostdb.org) and can be accessed using the dataset identifier PXD048118. Public data used in this study were listed below.

Processed data of human embryonic gut 10X scRNA-seq is from https://cellxgene.cziscience.com/collections/17481d16-ee44-49e5-bcf0-28c0780d8c4a.
Human gastrula Smart-Seq2 scRNAseq data is from E-MTAB-9388 (https://www.ebi.ac.uk/biostudies/arrayexpress/studies/E-MTAB-9388).
Human gonadal ovary 10X scRNA-seq data (wk11) is from GSE194266 (https://www.ncbi.nlm.nih.gov/geo/query/acc.cgi?acc=GSE194266).
Human gonadal ovary 10X scRNA-seq data (wks 7/9/10/13/16) is from GSE143380 (https://www.ncbi.nlm.nih.gov/geo/query/acc.cgi?acc=GSE143380).
Human embryonic sac model is from GSE134571 (https://www.ncbi.nlm.nih.gov/geo/query/acc.cgi?acc=GSE134571).
Human ESCs, pre-mesoderm cells, mesoderm cells, definitive endoderm cells, human PGCLCs, and human PGCs ChIP-seq data is from GSE159654 (https://www.ncbi.nlm.nih.gov/geo/query/acc.cgi?acc=GSE159654).
hPGCLC induction and hPGCLC culture methylome data are from GSE86586 (https://www.ncbi.nlm.nih.gov/geo/query/acc.cgi?acc=GSE86586) and GSE174485 (https://www.ncbi.nlm.nih.gov/geo/query/acc.cgi?acc=GSE174485), respectively.

Research involving human participants, their data, or biological material

Policy information about studies with human participants or human data. See also policy information about sex, gender (identity/presentation), and sexual orientation and race, ethnicity and racism.

Reporting on sex and gender	NA
Reporting on race, ethnicity, or other socially relevant groupings	NA
Population characteristics	NA
Recruitment	NA
Ethics oversight	NA

Note that full information on the approval of the study protocol must also be provided in the manuscript.

Field-specific reporting

Please select the one below that is the best fit for your research. If you are not sure, read the appropriate sections before making your selection.

☒ Life sciences ☐ Behavioural & social sciences ☐ Ecological, evolutionary & environmental sciences

For a reference copy of the document with all sections, see nature.com/documents/nr-reporting-summary-flat.pdf

Life sciences study design

All studies must disclose on these points even when the disclosure is negative.

Sample size	Sample size was not predetermined. The number of cells used for a statistical test was enough when comparing to previous studies reporting quantification of immunofluorescence signals(Ohta et al., 2017; Nagano et al., 2022). The number of genes used for statistical tests was determined by a biological criteria.
Data exclusions	For bulk RNA-seq, a sample derived from cells collected by inadequate FACS sorting gate was excluded retrospectively. For scRNA-seq by 10x chromium, low quality cells were excluded based on criteria described Methods section.
Replication	The exact number of replicates were indicated in the main text, figure legend or Methods section.
Randomization	For hPGCLC expansion culture experiments, the initial population of the expansion culture was randomly assigned by FACS sorting from gated cells. For immunofluorescence quantification analysis, images used for analysis were taken from randomly chosen locations.
Blinding	All experiments were not blinded. Investigators who performed experiments planned the sample allocation and culture conditions.

Reporting for specific materials, systems and methods

We require information from authors about some types of materials, experimental systems and methods used in many studies. Here, indicate whether each material, system or method listed is relevant to your study. If you are not sure if a list item applies to your research, read the appropriate section before selecting a response.

Materials & experimental systems

n/a	Involved in the study	
☐	☒	Antibodies
☐	☒	Eukaryotic cell lines
☒	☐	Palaeontology and archaeology
☒	☐	Animals and other organisms
☒	☐	Clinical data
☒	☐	Dual use research of concern
☒	☐	Plants

Methods

n/a	Involved in the study	
☒	☐	ChIP-seq
☐	☒	Flow cytometry
☒	☐	MRI-based neuroimaging

Antibodies

Antibodies used	rat anti-GFP, monoclonal Nacalai Tesque 04404-84

Antibodies used

mouse anti-UHRF1, monoclonal Millipore MABE308
goat anti-tdTomato, polyclonal Origene AB8181-200
mouse anti-AP2γ/TFAP2C, Santa Cruz sc-12762
mouse anti-DAZL, monoclonal Santa Cruz sc-390929
rabbit anti-DDX4 antibody, abcam ab13840
rabbit anti-5hmC antibody 39069 Active Motif
rabbit anti-5mC antibody SIGMA SAB5600040
rabbit anti p44/42 MAPK(ERK1/2) monoclonal CST 4695
rabbit anti phospho p44/42 MAPK(ERK1/2) monoclonal CST 4370
mouse anti alpha Tublin monoclonal SIGMA T9026
mouse anti-TRA-1-85 conjugated with Brilliant Violet 421, monoclonal BD Bioscience 563302
mouse anti EpCAM antibody conjugated with APC monoclonal BioLegend, 324208
rat anti CD49f (ITGA6) antibody conjugated with APC monoclonal BioLegend, 313624
rat anti CD49f (ITGA6) antibody conjugated with PE monoclonal BioLegend, 313611

Alexa Fluor 488 donkey anti-rat IgG Invitrogen A21208
Alexa Fluor 568 donkey anti-goat IgG Invitrogen A11057
Alexa Fluor 647 donkey anti-mouse IgG Invitrogen A31571
anti-rabbit IgG goat IgG conjugated with HRP SIGMA A6154
anti-mouse IgG sheep IgG conjugated with HRP SIGMA A5906

Validation

rat anti-GFP, monoclonal Nacalai Tesque 04404-84
Validation performed by the external collaborator was provided by the manufacturer (https://www.nacalai.co.jp/global/download/pdf/Epitope_Tag_Antibody.pdf). This antibody was used in previous studies(Yamashiro et al., 2018, Murase et al., 2020, Gyobu-Motani et al., 2023)

mouse anti-UHRF1, monoclonal Millipore MABE308
Validation was performed by the manufacturer using HeLa cells (https://www.merckmillipore.com/JP/ja/product/Anti-ICBP90-UHRF1-Antibody-clone-1RC1C-10,MM_NF-MABE308).

goat anti-tdTomato, polyclonal Origene AB8181-200
Validation was performed by the manufacturer using 293T cells expressing tdTomato (https://www.origene.com/catalog/antibodies/tag-antibodies/ab8181-200/tdtomato-goat-polyclonal-antibody).

mouse anti-AP2γ/TFAP2C, Santa Cruz sc-12762
Validation was not described by the manufacturer.(https://www.scbt.com/ja/p/ap-2gamma-antibody-6e4-4) This antibody was used in previous studies (Murase et al., 2020, Mizuta et al., 2022)

mouse anti-DAZL, monoclonal Santa Cruz sc-390929
Validation was not described by the manufacturer. This antibody was used in previous studies (Murase et al., 2020, Mizuta et al., 2022) (https://www.scbt.com/ja/p/dazl-antibody-e-6).

rabbit anti-DDX4 antibody, abcam ab13840
Validation was not described by the manufacturer.(https://www.abcam.co.jp/products/primary-antibodies/ddx4--mvh-antibody-ab13840.html) This antibody was used in previous studies (Mizuta et al., 2022)

rabbit anti-5hmC antibody, polyclonal 39069 Active Motif
This antibody was validated for MeDIP and dot blot analysis the by manufacturer (https://www.activemotif.com/catalog/details/39769.html).

rabbit anti-5mC antibody, monoclonal SIGMA SAB5600040
Validation was not described by the manufacturer, but was recommended to be used for ELISA, dot blot, immunocytochemistry, or immunohistchemistry (https://www.sigmaaldrich.com/JP/ja/product/sigma/sab5600040).

rabbit anti p44/42 MAPK(ERK1/2) monoclonal CST 4695
Validation was not described by the manufacturer, but can be applicable to western blotting (https://www.cellsignal.jp/products/primary-antibodies/p44-42-mapk-erk1-2-137f5-rabbit-mab/4695).

rabbit anti phospho p44/42 MAPK(ERK1/2) monoclonal CST 4370
Validation was not described by the manufacturer, but can be applicable to western blotting (https://www.cellsignal.jp/products/primary-antibodies/phospho-p44-42-mapk-erk1-2-thr202-tyr204-d13-14-4e-xp-rabbit-mab/4370).

mouse anti alpha Tublin monoclonal SIGMA T9026
Validation was not described by the manufacturer, but can be applicable to western blotting (https://www.sigmaaldrich.com/JP/en/product/sigma/t9026).

mouse anti-TRA-1-85 conjugated with Brilliant Violet 421, monoclonal BD Bioscience 563302
Validation was not described by the manufacturer, but can be applicable to flow cytometry (https://www.bdbiosciences.com/ja-jp/products/reagents/flow-cytometry-reagents/research-reagents/single-color-antibodies-ruo/bv421-mouse-anti-human-tra-1-85-antigen.563302).This antibody was used in previous studies (Murase et al., 2020)

mouse anti EpCAM antibody conjugated with APC monoclonal BioLegend, 324208
Validation was not described by the manufacturer, but can be applicable to flow cytometry (https://www.biolegend.com/de-at/cell-health/apc-anti-human-cd326-epcam-antibody-3758). This antibody was used in previous studies (Murase et al., 2020)

rat anti CD49f (ITGA6) antibody conjugated with APC monoclonal BioLegend, 313624

Validation was not described by the manufacturer, but can be applicable to flow cytometry (https://www.biolegend.com/de-de/products/brilliant-violet-421-anti-human-mouse-cd49f-antibody-8644?GroupID=BLG10323). This antibody was used in previous studies (Murase et al., 2020)

rat anti CD49f (ITGA6) antibody conjugated with PE monoclonal BioLegend, 313611
Validation was not described by the manufacturer, but can be applicable to flow cytometry (https://www.biolegend.com/fr-fr/products/pe-anti-human-mouse-cd49f-antibody-4108).

Alexa Fluor 488 donkey anti-rat IgG Invitrogen A21208
According to supplier's description, cross-reactivity to bovine, chicken, goat, guinea pig, hamster, horse, human, mouse, rabbit, and sheep serum proteins was reduced to the minimum level by affinity-based purification. Specificity of the antibody was increased by cross-adsorption. This antibody can be used for immunofluorescence (https://www.thermofisher.com/antibody/product/Donkey-anti-Rat-IgG-H-L-Highly-Cross-Adsorbed-Secondary-Antibody-Polyclonal/A-21208).

Alexa Fluor 568 donkey anti-goat IgG Invitrogen A11057
According to supplier's description, cross-reactivity to bovine, chicken, goat, guinea pig, hamster, horse, human, mouse, rabbit, and sheep serum proteins was reduced to the minimum level by affinity-based purification. Specificity of the antibody was increased by cross-adsorption. This antibody can be used for immunofluorescence (https://www.thermofisher.com/antibody/product/Donkey-anti-Goat-IgG-H-L-Cross-Adsorbed-Secondary-Antibody-Polyclonal/A-11057).

Alexa Fluor 647 donkey anti-mouse IgG Invitrogen A31571
According to supplier's description, cross-reactivity to bovine, chicken, goat, guinea pig, hamster, horse, human, mouse, rabbit, and sheep serum proteins was reduced to the minimum level by affinity-based purification. Specificity of the antibody was increased by cross-adsorption. This antibody can be used for immunofluorescence (https://www.thermofisher.com/antibody/product/Donkey-anti-Mouse-IgG-H-L-Highly-Cross-Adsorbed-Secondary-Antibody-Polyclonal/A-31571).

anti-rabbit IgG goat IgG conjugated with HRP SIGMA A6154
According to supplier's description, this is affinity-isolated antibody. This antibody can be used for western blot (https://www.sigmaaldrich.com/JP/en/product/sigma/a6154).

anti-mouse IgG sheep IgG conjugated with HRP SIGMA A5906
According to supplier's description, this is affinity-isolated antibody. This antibody can be used for western blot (https://www.sigmaaldrich.com/JP/en/product/sigma/a5906).

Eukaryotic cell lines

Policy information about cell lines and Sex and Gender in Research

Cell line source(s)	iPSC cell lines 585B1, 1390G3 and 1383D6 was provided by Dr. Keisuke Okita (CiRA, Kyoto, Japan). iPSC cell line NCLCN was purchased from XCell Science and licensed for academic use. M1-BTAG(585B1-BTAG) and F2-AGVT(1390G3-AGVT) lines were generated in previous studies (Sasaki et al., 2015, Yamashiro et al., 2018). M1-AGDT/AGVT(585B1-AGDT/AGVT) and F1-AGVT(NCLCN-AGVT) lines were generated in this study.
Authentication	iPSC cells were maintained in AK02N or AK03N which was developed for maintaining iPSC cell lines. iPSCs were authenticated by a typical morphology of human primed pluripotent stem cells. For TET1 KO iPSC cell line, each clone was authenticated by the specific indel pattern in the exon 6 as follows: 14 bp ins/1 bp del for KO#1, 28 bp del/20 bp del for KO#2.
Mycoplasma contamination	Cell lines used in this study were tested and confirmed to be negative for mycoplasma contamination .
Commonly misidentified lines (See ICLAC register)	No commonly misidentified lines were used in this study.

Flow Cytometry

Plots

Confirm that:

☒ The axis labels state the marker and fluorochrome used (e.g. CD4-FITC).

☒ The axis scales are clearly visible. Include numbers along axes only for bottom left plot of group (a 'group' is an analysis of identical markers).

☒ All plots are contour plots with outliers or pseudocolor plots.

☒ A numerical value for number of cells or percentage (with statistics) is provided.

Methodology

Sample preparation	Expanded hPGCLCs were treated with a 1:4 mixture of 0.5% trypsin–EDTA and PBS (−) at 37°C for 5 min. Alternatively, Accumax can be used instead. After the incubation, trypsin treatment was neutralized by adding advanced RPMI containing 10 % FBS, 0.1 mg/mL DNase1 and 10 uM Y27632. Alternatively, the expansion culture medium can be used instead. Cell suspension was directly subjected after filtration by a strainer. DAPI was added to the cell suspension if needed.

Instrument	FACS Aria 3
Software	FACS Diva
Cell population abundance	Cell sorting by FACS Aria 3 was performed in "purity mode" to ensure the purity of sorted cell population.
Gating strategy	585B1-AGDT, NCLCN-AGVT<br>First, cell debris was excluded by gating on FSC/SSC plot. Second, single cell population was gated on FSC-height/FSC-width plot and SSC-height/SSC-width plot. Third, if needed, DAPI(+) dead cells were excluded by gating on DAPI/FSC plot. Resultant single live cells were used for the reporter expression analysis.<br><br>585B1-BTAG, TET1KO#1, TET1KO#2<br>First, cell debris was excluded by gating on FSC/SSC plot. Second, single cell population was gated on FSC-height/FSC-width plot and SSC-height/SSC-width plot. Resultant single live cells were used for the reporter expression analysis. BT(+) cells and AG(+) cells were gated independently on BT/APC or AG/APC plot, respectively.<br><br>585B1-BTAG (TRA-1-85 expression analysis)<br>First, cell debris was excluded by gating on FSC/SSC plot. Second, single cell population was gated on FSC-height/FSC-width plot and SSC-height/SSC-width plot. Resultant single live cells were used for TRA-1-85 expression analysis. BT(+)AG(+) cells were gated on BT/AG plot. Then, non-BTAG cells were plotted on FSC/SSC plot.<br><br>1383D6 (EpCAM/ITGA6 expression analysis)<br>First, cell debris was excluded by gating on FSC/SSC plot. Second, single cell population was gated on FSC-height/FSC-width plot and SSC-height/SSC-width plot. Resultant single live cells were used for EpCAM/ITGA6 expression analysis.

☒ Tick this box to confirm that a figure exemplifying the gating strategy is provided in the Supplementary Information.
